# Educational Psychology-Based Strategy for Instrumental Music Teaching in Normal College

**DOI:** 10.3389/fpsyg.2021.657788

**Published:** 2021-10-25

**Authors:** Yang Li

**Affiliations:** School of Music, Shandong Normal University, Jinan, China

**Keywords:** normal college, instrumental music teaching, musical teaching strategy, teaching mode, music teaching methods

## Abstract

The study is intended to explore the teaching mode of instrumental music teaching in normal college, so as to have qualified instrumental music teaching talents. Based on relevant content of educational psychology, the current instrumental music teaching model of normal college should be reformed in terms of curriculum content, teaching mode, and teaching methods, and a more systematic and standardized education model should be established. As per Orff’s music teaching method, a music recommendation model is established based on the convolutional neural network model to provide students with a positive and happy learning environment for instrumental music, and music materials that meet their personal preferences and performance level through user data. The outcomes show that the designed music recommendation model has a music recommendation accuracy rate of 0.3 and a recall rate of 0.29 when the recommendation list is 30, which conforms to the general rules of the music recommendation system. The study is expected to provide reference for establishment of a standardized and systematic instrumental music teaching strategy in normal college.

## Introduction

The goal of musical teaching in normal college is to train students to be music talents who have good music literacy and adapt to the teaching tasks of primary and secondary schools, and the training process is highly professional. Therefore, in training process in normal college, only imparting professional knowledge and related teaching skills can’t meet the teaching needs. Students should consolidate and apply the knowledge they have learned so as to gain valuable teaching experience. Instrumental playing is the foundation of instrumental music teaching, and this process can cultivate students’ cultural aesthetics (Anglada-Tort and Sanfilippo, 2019). Traditional instrumental music teaching is no longer suitable for current needs due to old teaching content, single teaching methods, without integration with related fields, and excessive pursuit of performance skills. Therefore, it is imperative to reform instrumental music teaching. Students in normal college need to have educational practice in primary and secondary schools, adapt to the teaching environment of the school, and experience the change of the role, which provides a basis for personal professional development (Burak, 2019).

Rickels et al. (2019) studied the motivation and influence of interest and disinterest in the music teaching profession. They used the principal component discriminant analysis method to compare three occupational categories of music teaching, other music, and other non-music. Experimental results showed that, the correct classification rate of this method was 69.8%. One factor can distinguish two music groups from the non-music group, and the other three factors can distinguish the partial music group from non-music group. Therefore, the choice of profession seems to be diversifying, which cannot be predicted through a single factor (Rickels et al., 2019). Schiavio et al. (2020) studied 11 music expert teachers based on their individual and collective environment teaching practice. They adopted a basic theory-based method to identify two interrelated themes in the original data: teaching problems and professional development. In these two themes, the concept of “presence” was a decisive feature for the comparison. In a collective environment, teachers have a low sense of presence, while higher expectations of people and higher requirements (cognition, teaching, etc.) of learners will cause teachers to participate more in the teaching. Experimental results showed that, in a collective environment, teachers can also be learners, reducing their own sense of presence. Students’ sense of responsibility and learning ability were fostered by establishing a hybrid teaching expansion system (Schiavio et al., 2020). Abramo and Campbell (2019) surveyed 12 senior students majoring in music education and preschool education, about their expectations and beliefs about the relationship between them and their co-teachers before their teaching experience. The results of the questionnaire survey showed that, the participants hope that the co-teachers can be their friends. The senior students thought that, their relationship with the co-teachers was a standardized practice; but they also hoped that, the co-teachers can provide emotional support and share personal stories. Therefore, based on these findings, teachers may give co-teachers direct educational guidance, with philosophical guidance as the center, and envisage teaching experience beyond the one-to-one guidance relationship (Abramo and Campbell, 2019). Vasil et al. (2019) studied the popular music teaching in music teaching, based on the theory of educational psychology. The experiment found that, the popular music teaching method can enable students to have the ability to think critically. Teachers can create by interacting with students, to teach music knowledge (Vasil et al., 2019). Menz et al. (2020) studied pre-service teachers’ misunderstanding of educational psychology, which may pose a threat to actual teaching goals. Through a questionnaire survey on the misunderstandings about educational psychology among pre-service teachers, it was found that, only a few teachers can change their attitude toward misunderstandings. Therefore, misunderstandings of educational psychology are common among pre-service teachers (Menz et al., 2020).

To the best of our knowledge, the research on instrumental music teaching in normal college mainly focuses on the interaction between teachers and students. Regarding low teaching efficiency and low quality of teaching in traditional instrumental music teaching in normal college, it is necessary to cultivate qualified talents for instrumental teaching in normal colleges, to enhance students’ enthusiasm for learning instrumental music. In the study, the instrumental music teaching strategies in normal college are analyzed based on educational psychology, and reform is recommended from five aspects of the learning theory, teaching elements, curriculum elements, teaching process, and teaching mode, so as to have a more systematic instrumental music teaching method. Further, combined with Orff’s music teaching method, students are provided with a positive and encouraging learning environment, and suitable musical materials are recommended based on the CNN, so that students can practice in an environment that matches their music preferences. In conclusion, the Orff’s music teaching method is combined with CNN to provide students with a pleasant instrumental music learning environment, aiming to promote the overall development of instrumental music education, establish a systematic and standardized instrumental music teaching method, and comprehensively improve teaching quality. Besides, it is expected to have primary and secondary school music teachers with good performance skills, mental health, and a solid theoretical foundation.

## Materials and Methods

### Educational Psychology-Based Instrumental Music Teaching Reform

Instrumental music teaching is a very important music teaching mode, which can stimulate students’ enthusiasm for learning instrument, and cultivate students’ creative thinking and perfect personality. Instrumental music teaching reform in normal college is affected by the traditional music teaching model, and lacks relevant theoretical support and guidance. Therefore, the reform process is restricted. From the perspective of educational psychology, the reform of instrumental music teaching mode should take account into students’ cognitive styles, and the connection between teaching mode and teaching strategy should be considered. Cognitive style is the habitual attitude that individuals have when processing and organizing information in cognitive activities such as feeling and understanding (Burwell et al., 2019).

The relevant background knowledge of instrumental music should be added to instrumental music teaching, such as the origin of musical instruments, development history, art form, cultural background, composer and creative style, representative composition analysis, and repertoire appreciation can help students understand the cultural deposits of instrumental music and improve the overall artistic quality of students. As a result, their professional understanding is more comprehensive and their performance ability is improved (Taft et al., 2020). Besides the traditional instrumental music works, students should also learn world folk music works and modern works. By playing different styles of works, they can understand the characteristics of different styles of writers and genres, and enhance their aesthetic ability (Öztuğ and Saldun, 2020). According to teaching characteristics in normal college, content related to music teaching in primary and secondary schools is introduced for learning.

Traditional instrumental music teaching mostly adopts one-to-one teaching method, which has low efficiency. What’s more, the teaching quality can’t be guaranteed, because students from different majors have different learning abilities (Julia et al., 2020). There are two types of courses for students: elective courses and compulsive courses. With the deepening of the teaching, the focus has changed. From the single instrumental music performance at the beginning, students begin to have practical training courses and rehearsal courses (Kim, 2019). Hence, the corresponding teaching mode and teaching methods need to be adjusted to better meet the teaching requirements. For different curriculum models, different teaching methods should be adopted. For students majoring in instrumental music, a one-to-one teaching method should be adopted to conduct targeted observations on students who already have certain performance abilities and skills, and solve different problems in the performance of students (Bayley and Waldron, 2020). At the same time, teachers should formulate a personalized learning plan and choose a piece of music suitable for students’ level, to enhance students’ performance skills and inspire students to understand music more deeply.

For students who take instrumental music as elective courses, one-to-many teaching method should be adopted. Because they mostly major in vocal music or piano and other subjects, they haven’t learned instrumental music before. The elective courses can help students master instrumental performance and band rehearsal, thus becoming a more comprehensive talent (Matthews and López, 2020). Therefore, the focus of teaching is on the basic knowledge of instrumental music and basic performance skills. By understanding the history of musical instruments and mastering the basic playing methods of musical instruments, students therefore improve their personal performance ability and group ensemble ability (Cho, 2019). In instrumental music teaching, great importance should be attached to the training of students’ practical skills. Students should apply the knowledge of instrumental music they have learned to practice, and discover problems in learning through practice. It is also feasible to organize open classes and concerts, so that students can have training, thereby enhancing their actual performance ability. Therefore, in the instrumental music teaching course, it is necessary to combine specific teaching modes, establish a more scientific and complete instrumental music teaching system on the basis of the original teaching, and integrate actual skill training to improve the teaching level and quality (Palkki and Sauerland, 2019).

### Information Technology in Instrumental Music Teaching in Normal College

Accompanied by social informatization, the introduction of information technology in teaching has greatly challenged the traditional teaching. Therefore, it is imperative to promote informatization of instrumental music teaching in normal colleges, so as to establish modern educational technology with computer technology as the core. With the advancement of technology, teachers need to combine computer technology to collect, organize, and display various instrumental music teaching materials, and use them in courses. The application of computer technology can stimulate teachers’ creativity and enthusiasm for teaching, and can also enhance students’ enthusiasm for learning. The use of computer technology to synthesize piano accompaniment and harmony accompaniment. Then, students can ensemble with accompaniment, which cultivates students’ teamwork ability and awareness. Students should be encouraged to participate in various performance activities to accumulate performance experience and discover their own shortcomings, so as to improve their ability. Students can also learn instrumental music by listening to related lectures or concerts. Therefore, multiple teaching methods can enrich the teaching content of instrumental music teaching in normal college, improve students’ artistic appreciation ability, expand students’ innovative thinking, and elevate students’ instrumental performance skills (Zhang et al., 2020).

Since normal college aims to train students to be teachers, students should not only focus on instrument skills, but also learn teaching methods, and combine instrumental music teaching, educational psychology, and instrumental music ensemble with instrumental music courses. Instrumental music teaching elaborates on the basic content, process, rules, and methods of musical instrument teaching (Savage et al., 2020), which can help students have a comprehensive and in-depth understanding of the relevant elements of learning, guide them to grasp the rules, and improve their playing level. The instrumental music teaching method can also provide theoretical guidance for students’ future teaching work. In the courses of instrumental performance and instrumental music teaching, students learn the teaching methods while learning performance skills. In this learning process, students learn certain instrumental performance skills and deepen their understanding of musical instruments. At the same time, they learn how to teach by observing the teaching methods of teachers (Freer and Evans, 2019).

Educational psychology theory is to analyze the relationship between behavior process, psychological process, and teaching process. Relevant psychological theories are instrumental in explaining students’ music behavior and music experience, improving students’ performance skills by enhancing their psychological quality. Instrumental music courses are an integral part of music teaching in normal college, which is expected to strengthen students’ understanding of collective instrumental performance by perfecting students’ performance and teamwork skills. How to make students have a pleasant learning attitude in teaching is the focus of educational psychology. The Orff’s music teaching method is to establish a positive and encouraging music learning environment for students, so that students can have a systematic instrumental music learning method. Its secondary aim is to have qualified primary and secondary school music teachers.

### Orff’s Music Teaching Method Combined With CNN in Instrumental Music Teaching in Normal College

Traditional instrumental music teaching emphasizes the teaching of knowledge and skills, and the static teaching method is adopted. However, due to the characteristics of music itself, students are required to communicate with the audience through music. Therefore, the Orff method is applied to optimize the teaching strategies of instrumental music in normal college. Teaching strategy includes five parts: learning theory, teaching elements, curriculum elements, teaching process, and teaching mode. Teaching elements include teaching objectives, content, methods, and resources (Ros-Morente et al., 2019). Curriculum elements include teachers, students, teaching materials, and environment. When designing teaching strategies, it is necessary to consider different teaching elements, analyze the role of teachers in teaching, and think about the teaching methods and resources needed to better serve students. Orff’s music teaching method was created by German music educator Carl Orff. Orff believes that successful music teaching is positive and encouraging, allowing students to feel the value of their own development (Cowie, 2020). Its purpose is to show the diversity and richness of music, and to encourage students to learn in activities. As a result, students obtain things beyond music learning, such as imagination, teamwork ability, self-learning ability, and creativity (Kellems et al., 2019). Therefore, the use of Orff’s music teaching method in the instrumental music teaching in normal college makes teachers focus on the quality of teaching and the cultivation of students’ playing skills.

**FIGURE 1:**
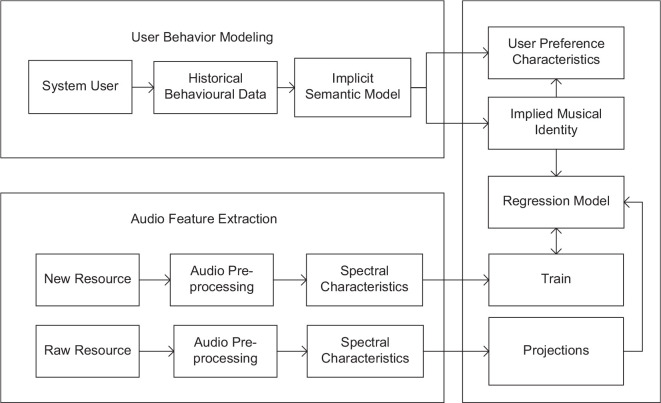
CNN-based music recommendation model.

As per Orff’s music teaching method, students are provided with a positive and encouraging music learning environment to raise their learning interests and reduce teachers’ pressure in class. Specifically, a music recommendation algorithm is established based on CNN to predict the hidden features of the music materials, and the low-dimensional vector of the music features is then obtained. Finally, the practice materials are recommended according to the user’s preference features. The music recommendation process is shown in Figure 1.

The music recommendation model includes an audio feature extraction module, a recommendation algorithm module, and a user modeling module. The audio feature extraction module can perform preprocessing and feature extraction on music. The recommendation algorithm can predict the potential characteristics of music according to the regression model and combine user preferences to obtain the matching degree between the user and the music, and generate a list of music that the user may like. The user modeling module can collect students’ choices and build user preference feature models. The whole model is divided into two parts: regression training and prediction recommendation (Bertka et al., 2019).

In music information retrieval, it is necessary to extract the characteristics of the audio to reflect the essential information of the music to distinguish different music (Vasil, 2019). Audio features can be divided into two types, namely, time domain features and frequency domain features. In the study, short-time Fourier transform is used to extract audio features. For non-stationary non-periodic audio signals whose frequency spectrum changes continuously over time, they can be changed from the time-domain signals to frequency-domain signals. The frequency component of the signal can be seen intuitively, but without the time-domain information, the relationship between the frequency distribution and time can’t be obtained (Schedl, 2019). The Short Time Fourier Transform (STFT) is a two-dimensional representation method of time domain and frequency domain. Audio is a signal that changes slowly with time. The STFT can divide a long-term signal into frames, and perform Fourier transform on each frame to express the characteristics of the audio signal at a certain time (Salvador, 2019). The equation for performing short-time Fourier transform on the source signal *x*(*t*) is as follows.


(1) $$STFT\left\{x\left(t\right)\right\}\left(\tau ,\theta \right)=\int_{-\infty }^{\infty }x\left(t\right)w\left(t-\tau \right)e^{-j\omega t}dt$$

*w*(*t*) is a window function; *STFT*{} represents the STFT; *e*^−*j*ω*t*^ represents the negative frequency domain; *t*, τ, and θ are parameters in STFT. The discrete signal is expressed as follows.


(2) $$STFT\left\{x\left(n\right)\right\}\left(m,\omega \right)=\sum_{n=-\infty }^{\infty }x\left(n\right)w\left(n-m\right)e^{-j\omega n}$$

*w*(*n*−*m*) represents the window function sequence, and *n* represents the discrete time signal. After the transformation of the audio signals, the amplitude and phase information of different frequencies are obtained (Gunawan and Suhartono, 2019). The phase information can be converted into a spectrogram of amplitude information. The equation of transformation between short-time power spectrum and STFT is as follows.


(3) $$SP_{x}\left(m,\omega \right)=\left|X\left(m,\omega \right)\right|$$

The spectrogram can be regarded as a two-dimensional signal image which is formed by superimposition of each frame of the STFT. Then, a spectrogram containing two-dimensional signals of the time domain and frequency domain is obtained.

### The Music Recommendation Model Based in Convolutional Neural Network

**FIGURE 2:**
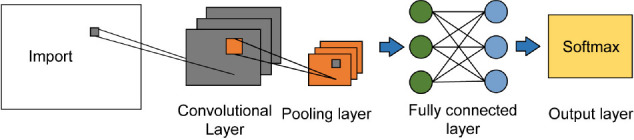
The structure of CNN.

The neural networks are used to perform feature analysis on spectrograms of different types of music, together with spectrum images, and the music in the data set is classified according to music genre tags. CNN is a feedforward neural network that includes convolution operations and deep structures. Its basic structure includes the input layer, the convolution layer, the pooling layer, the fully connected layer, and the output layer. As shown in Figure 2, using local connections avoids the problem of information loss under a large number of parameters (Du et al., 2019). First, the input layer reads the information matrix of the input data; then it outputs to a convolutional layer with multiple feature surfaces. Each feature surface has multiple neurons, and the input of each neuron node is the result of the previous network block. The depth information of the data can be extracted using the convolution block in the convolution layer to obtain higher-dimensional feature information. The pooling layer reduces the dimensionality of the high-level features output by the convolutional layer. The neurons between the layers in the fully connected layer are fully connected, and the feature information obtained by the convolutional layer and the pooling layer is classified. The output layer uses the Softmax function to classify features and output the results. The probability distribution of each category is calculated, the category with the highest probability is the category of the test sample (Zhang and Yang, 2019).

Compared with artificial neural networks, CNN introduces the concepts of local perception, weight sharing, and downsampling, which greatly improves the performance of the network. Local perception means that each neuron in the CNN only connects to some neurons in the next layer, thereby reducing the weight parameters. Therefore, the connection between local information is close, and the correlation with farther information is weak. Only the neighboring information needs to be perceived, which can greatly reduce the computing time (Alkalay and Dolev, 2019; Wen, 2020). As the number of layers increases, local feature extraction can be continued on the feature information extracted by the previous layer to obtain global information of the input information. In CNN, weight parameters or convolution kernels are shared between each group, instead of each connection having its own weight. The convolution kernel has a specific feature in a certain area, then this convolution kernel can also be used in other areas with the same feature. CNN uses downsampling technology to compress the input information, reduce the total volume of input data, and reduce the over-fitting phenomenon caused by too many weight parameters. In the meanwhile, because the data space size is compressed, the amount of calculation of CNN is greatly reduced and the calculation speed is accelerated. Table 1 shows CNN parameter setting.

**TABLE 1:** CNN parameter setting.

Number of network layers	2-layer	Learning rate	0.1
Convolution kernel size	5 × 5	Loss function	Quadratic mean square function
Optimization algorithm	Adagard algorithm	Mini-batch	30
Activation function	Relu function	Initial weight	1.5

### The Database Used and the Experimental Environment

A large amount of data is needed to train and test the designed music recommendation model, but due to copyright restrictions, most databases do not directly provide audio files. Therefore, to train and test the designed model, the audio files used in the instrumental music teaching in normal college are collected to establish a data set containing audio files and user behavior records, as well as different users’ song playback records. The data is preprocessed to obtain a data set that meets the experimental requirements. The data set contains 700 common practice songs downloaded from the website. Among them, 400 songs are used as the training set, and the remaining 300 songs are used as the test set. Fifteen students majoring in instrumental music teaching of a normal college are selected as the research subjects, with each student’s song playing in 60 days recorded to obtain a data set of users and music playing times (Solanki and Pandey, 2019; Deng et al., 2021). The accuracy rate and recall rate are used as evaluation indexes. Table 2 is the experimental environment setting.

**TABLE 2:** The experimental environment.

System	Win10	RAM + internal storage	16 GB + 512 GB
CPU	i7-7700	Software	Matlab R2015a_x64
GPU	GeForce GTX 960	Acquisition equipment	Zoom H6+Rode NTG3

## Results and Discussion

**FIGURE 3:**
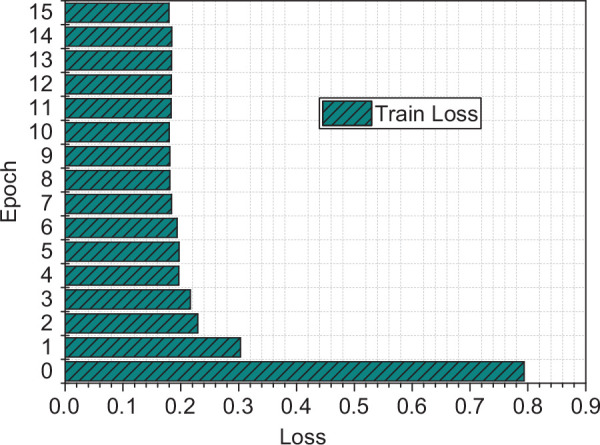
The loss curve of the trained model.

The mean square error (MSE) is used to establish the loss function. Then, the training set built is used to train the CNN model, and Figure 3 are the experimental results.

It is evident that as the number of iterations increases, the loss error of CNN begins to decrease rapidly. When the number of iterations reaches 10, the error drops to 0.128 and then tends to converge. According to the loss curve, the training of the model meets the expected effect, which can well test the predictive ability of the system.

**FIGURE 4:**
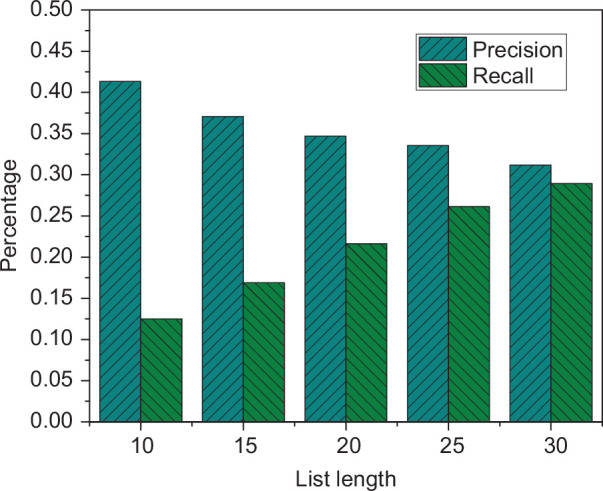
The recommendation results under different list length.

To verify the applicability of the music recommendation algorithm, the recommendation accuracy under different recommendation list lengths is tested. The length of the recommended list is set to 10, 15, 20, 25, 30, and the experimental results are shown in Figure 4.

It is evident from Figure 4 that as the length of the recommendation list increases, the accuracy rate is continuously decreasing, and the recall rate is continuously increasing. When the recommended list length is 10, the accuracy rate is about 0.41, and the recall rate is about 0.128. When the recommended list length increases to 30, the two are 0.3 and 0.29, respectively.

**FIGURE 5:**
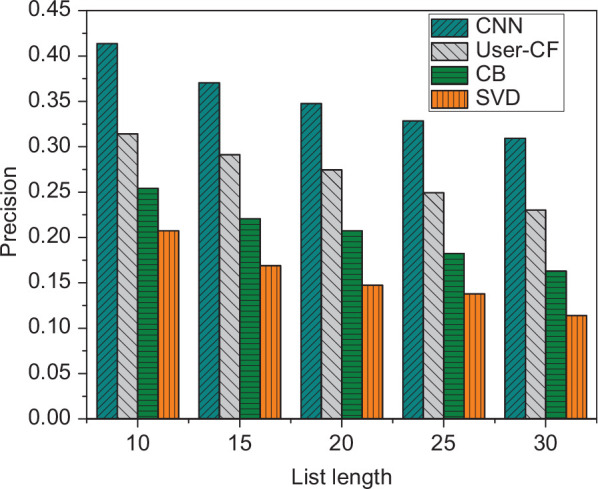
Recommendation accuracy of different algorithms.

To verify the effectiveness of the recommendation algorithm, the existing data set is used to conduct comparative experiments on different recommendation algorithms. The experimental results are shown in Figure 5.

It is evident from Figure 5 that under the same length of the recommendation list, the accuracy of the recommendation algorithm designed in this study is the highest. The reason may be that the algorithm designed in this study studies the interaction between users and music, so that CNN can better learn user habits.

By incorporating the educational psychology into the instrumental music teaching of normal college, the instrumental music teaching methods, music psychology, and instrumental ensemble will be integrated to help students better master the knowledge of instrumental music and gain practical experience, providing a foundation for future teaching work (Chen, 2020; Deng et al., 2021). In addition, the introduction of CNN provides students with an active instrumental learning environment. Orff’s music teaching method is referred to make students interested in learning during the learning process. The accuracy rate of the Western music recommendation model based on the CNN algorithm is as high as 96.5% (Chen, 2019), but the model designed in this study has a wider scope of application and a higher overall recommendation accuracy rate, creating a new teaching mode of instrumental music in normal college. The pre-service education is the first step of the professional development for normal college students. Through educational practice, students can experience teaching work in the front line, accumulate teaching experience, and cultivate professional awareness and responsibility. Secondly, through the educational practice, they experience role-changing from students to teachers, so that they can face different teaching situations (Numanee et al., 2020). Finally, they can independently engage in first-line instrumental music teaching jobs by using their theoretical knowledge and educational methods to perceive and reflect on the teaching situations they encounter.

## Conclusion

To explore the teaching strategies of instrumental music teaching in normal college and enhance students’ enthusiasm for learning instrumental music, the teaching method reform of instrumental music teaching in normal college is recommended from many aspects based on educational psychology, and a more systematic and standardized method of instrumental music teaching is established. Furthermore, the Orff music teaching method is combined with the CNN to establish a music recommendation model to provide students with practice materials that match their personal preferences and performance levels, thereby enhancing students’ enthusiasm for instrumental music learning. The experimental results show that the designed music recommendation model can provide students with practice materials in line with the students’ interest. However, some shortcomings should be noted in this study. The data set used for training is small, and the number of users used is not enough to train the model well. Therefore, an expanded sample size id needed in subsequent research to improve the predictive performance of the system.

## Data Availability Statement

The raw data supporting the conclusions of this article will be made available by the authors, without undue reservation.

## Ethics Statement

The studies involving human participants were reviewed and approved by the Shandong Normal University Ethics Committee. The patients/participants provided their written informed consent to participate in this study. Written informed consent was obtained from the individual(s) for the publication of any potentially identifiable images or data included in this article.

## Author Contributions

The author confirms being the sole contributor of this work and has approved it for publication.

## Conflict of Interest

The author declares that the research was conducted in the absence of any commercial or financial relationships that could be construed as a potential conflict of interest.

## Publisher’s Note

All claims expressed in this article are solely those of the authors and do not necessarily represent those of their affiliated organizations, or those of the publisher, the editors and the reviewers. Any product that may be evaluated in this article, or claim that may be made by its manufacturer, is not guaranteed or endorsed by the publisher.
